# Social Impacts of the American Red Cross (ARC) Disaster Interventions: A Scoping Review

**DOI:** 10.7759/cureus.55265

**Published:** 2024-02-29

**Authors:** Mishayla A Harve, Dan Li

**Affiliations:** 1 Ivan Allen College of Liberal Arts, Georgia Institute of Technology, Atlanta, USA; 2 College of Medicine, Yale University, New Haven, USA

**Keywords:** american red cross, disaster, emergency, hurricane katrina, shelter

## Abstract

The American Red Cross (ARC) self-reports post-disaster efforts annually, potentially biased for public image. The lack of formal reviews of ARC interventions' social impacts further exacerbates the issue. This scoping review aims to address both issues by summarizing and evaluating the social impact of ARC’s national disaster interventions. As a secondary objective, this review will also provide important information to guide ARC and other organizations in truly fulfilling their missions.

The inclusion criteria involve participants of all age groups, marginalized communities, displaced individuals, and ARC disaster responders. Opinionated statements, except for anecdotes, were excluded. This review involves all United States (US) areas that have been affected by disasters and required assistance from ARC. With no search limits, all evidence was searched on PubMed from July to August 2023. Two stages of screening were conducted by two independent reviewers: title/abstract screening and full-text screening. During each stage, each paper underwent a quality assessment. Disagreements in each stage were resolved before proceeding to the next stage.

Through 22 academic papers, the review outlines key themes in ARC comprehensive interventions on disaster preparedness and on-site disaster interventions. However, research gaps were found in ARC recovery interventions, especially their impact on evacuees.

This investigation found that ARC makes general efforts to address the needs of communities they assist before, during, and after disasters. Because the results suggest that ARC is moving in the correct direction in reducing the nationwide harm disasters cause, ARC's social impact on studied populations is mostly positive.

## Introduction and background

Introduction

The American Red Cross (ARC) was founded in 1881 by Clara Barton to aid those injured in war [1]. Generations later, the mission of the ARC expanded to encompass the alleviation of human suffering as an integral part of the broader volunteer network known as the Red Cross. As a humanitarian organization, ARC provides many nationwide services and has become a pillar for disaster relief interventions. Working most commonly during hurricanes, floodings, earthquakes, and fires, ARC provides aid, supplies, healthcare, and other resources to those affected by local crises [1].

Amidst its impactful efforts, ARC faces a challenge when it comes to assessing the impact of its disaster interventions. While ARC self-reports the effect of their continued annual efforts [2] after the disaster, the reports pose a high potential for sampling bias to maintain a public image [1]. Moreover, the scarcity of formally published review papers examining the short- and long-term impacts of national ARC interventions from social perspectives further compounds the issue. Without conclusive and impartial research on ARC’s disaster efforts, it could be argued that the organization is less aware of how to improve for future emergencies.

Therefore, the main objective of this paper is to identify and evaluate the impact of national disaster interventions by the ARC on affected populations, which will be conducted by looking at reports where ARC disaster relief was employed nationally. Additionally, there is a secondary objective. This investigation will not only enhance society’s understanding of ARC efforts but also provide important information to guide ARC and other organizations in truly fulfilling their missions.

Methods

Review Question

What are the social impacts (the implications of the accessibility of ARC disaster intervention resources) of national disaster interventions by the ARC on affected vulnerable populations?

Eligibility Criteria

Participants: The participants studied involved populations affected by disasters. This includes all age groups and marginalized communities (individuals with disabilities and individuals living at or below the poverty line). The participants also include those who have been forcibly displaced due to disaster (displaced individuals, migrants, and refugees) and those who assist in these crises (volunteers, emergency responders, and disaster responders).

Concepts: The main concept of ARC's involvement is the accessibility of its disaster intervention resources. The following disaster intervention resources are divided into three categories: disaster preparedness, on-site disaster interventions, and disaster recovery. The papers included in this review span from the creation of ARC to the present day.

Context: The context in this review involves all areas within the United States (US) that have been drastically affected by a man-made or natural disaster or heavily aided by ARC during the disaster emergency.

Types of sources: The types of sources used in this review include: surveys, pilot studies, cohort studies, systematic reviews, descriptive studies, literature reviews, anecdotes, observational, mixed methods, cross-sectional, and surveillance studies.

Exclusion Criteria

Papers explicitly excluded from this review will consist of opinionated statements, regardless of whether they reference an ARC disaster intervention. However, personal anecdotes are exempt from the exclusion criteria due to the valuable insights they provide. Other characteristics for excluding records include papers that evaluate outcomes unrelated to ARC's disaster interventions, fail to comprehensively measure or assess the social impact on affected populations, or do not involve the affected populations at all. Also, if the full text could not be accessed, the paper was excluded from this review. There are no restrictions on the time frame: papers included in this review span were published since the creation of ARC to the present day. The proposed scoping review will be conducted per the JBI methodology for scoping reviews [3].

Search Strategy

A single database search of PubMed was undertaken to identify articles on the topic, using keywords such as "American Red Cross" and "disaster." The text words contained in the titles and abstracts of relevant articles and the index terms used to describe the articles were used to develop a full search strategy:

(“American Red Cross”[Title/Abstract]) AND (“disaster”[Title/Abstract] OR “earthquake”[Title/Abstract] OR “tsunami”[Title/Abstract] OR “tropical storms”[Title/Abstract] OR “flood”[Title/Abstract] OR “epidemic”[Title/Abstract] OR “storm”[Title/Abstract] OR “mass violence”[Title/Abstract] OR “pandemic”[Title/Abstract] OR “attack*”[Title/Abstract] OR “emergency*”[tw] OR “international aid*”[tw] OR “migration*”[tw] OR “refugee*”[tw] OR “conflict*”[tw])

Study of Evidence Selection (Screening)

Following the search, 22 identified citations were collated and uploaded into a spreadsheet, and duplicates were removed. Two independent reviewers conducted two stages of screening: title/abstract screening and full-text screening. In addition to evaluating adherence to the inclusion and exclusion criteria, a quality assessment was also performed by both reviewers for each paper during each round of screening. The quality assessment was done in two stages. In the first quality assessment stage, the titles and abstracts of each record were screened to see if they met the eligibility criteria and rejected the exclusion criteria. If not, the record was excluded. In the second quality assessment stage, the full text of all accepted papers from the previous stage was screened in the same manner as the first stage.

Disagreements in the title/abstract stage of screening were resolved before proceeding to the full-text screening, and disagreements in the full-text screening were resolved before continuing with the paper.

Data Extraction

Data was extracted from papers included in the scoping review by two or more independent reviewers using the matrix method. A table was designed to include all the variables to aid in the extraction of each paper. The data extracted included specific details about the participants, concept, context, study methods, and key findings relevant to the review question.

## Review

Data analysis

**Figure 1:**
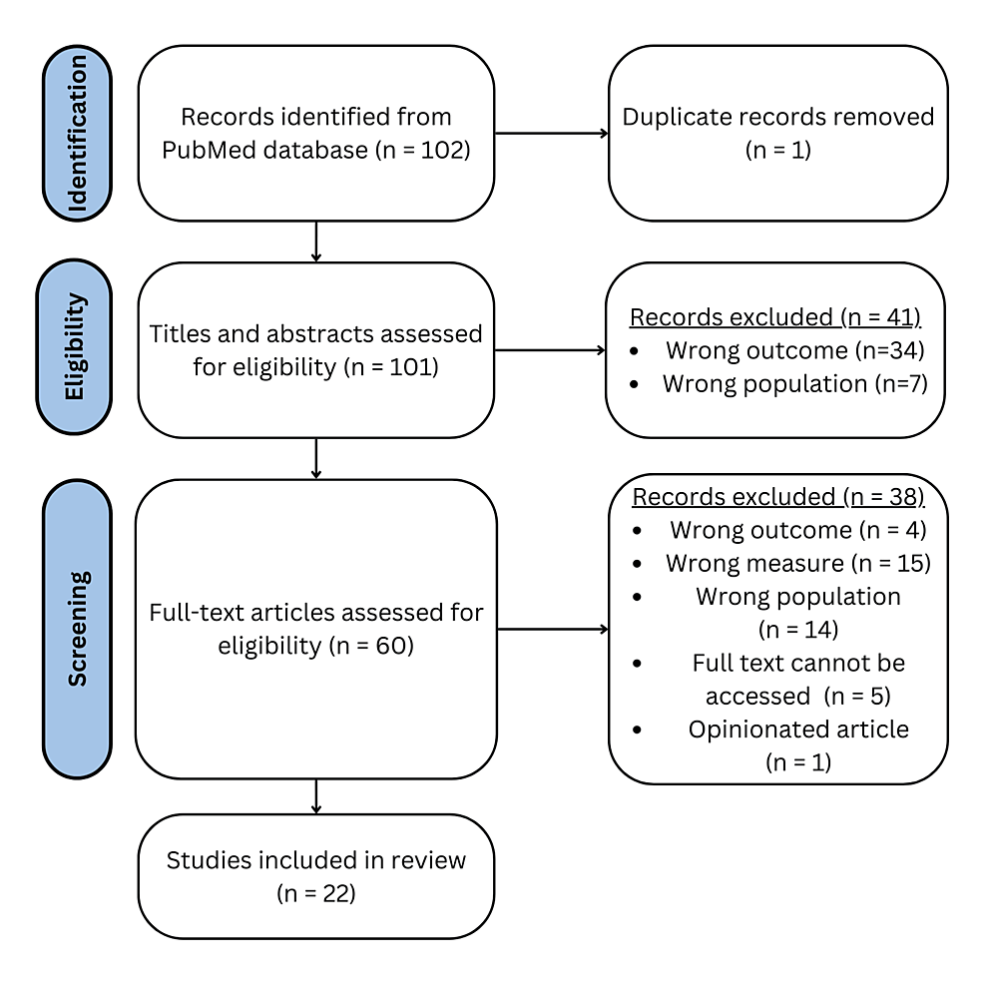
The PRISMA flowchart outlining the screening processes used The Preferred Reporting Items for Systematic Reviews and Meta-Analyses (PRISMA) flow diagram illustrates the search, screening, and final study inclusion. Records with the "wrong outcome" were studying something unrelated to ARC's disaster interventions. For example, a study assessing ARC's biomedical innovation in blood donations would have been excluded because it had the "wrong outcome." Records with the "wrong measure" did not comprehensively measure the social impact of the participating populations OR no useful inferences could be derived from the study. For instance, a study could have only briefly mentioned statistics of how a select intervention affected an eligible population but would be excluded because nothing "useful" could have been extrapolated from the record due to its oversimplification. Lastly, excluded records with "wrong population" only included populations that did not satisfy the eligibility requirements, such as blood donors.

The following Preferred Reporting Items for Systematic Reviews and Meta-Analyses (PRISMA) flowchart summarizes the screening results, revealing the number of papers qualified for each subsequent screening stage (Figure 1) [4]. The initial search utilized the PubMed search strategy, resulting in 102 articles. Sixty papers passed the first stage of screening. Of the 60 papers, 22 passed the second stage of screening.

Table 1 summarizes the characteristics of all the studies that will be included in the review. All of the papers have been grouped into three thematic categories: disaster preparedness, on-site disaster interventions, and long-term disaster recovery, as identified in the last column of the table.

**Table 1:** Final paper characteristics This table summarizes the characteristics of all the studies [5-26] that will be included in the review. BSN: bachelor of science in nursing, ARC: American Red Cross, N/A: not available, CBDP: community-based disaster preparedness, PsySTART: Psychological Simple Triage and Rapid Treatment, PTSD: post-traumatic stress disorder

PMID	Title	Study Type	Location Studied	Population Studied	Intervention(s) Studied	Outcome(s) Studied
29054103 [5]	Preparedness education in the baccalaureate nursing curriculum: an authentic clinical experience [5]	Pilot study	N/A	Nursing program	Preparedness education for nursing	Awareness of disaster preparedness procedures of nursing students in BSN program partnering with ARC
26369629 [6]	Emergency preparedness and disaster response: there’s an app for that [6]	Systematic review	N/A	N/A	Disaster response app	Analyzing the price and features of several emergency/disaster response apps, including applications by the ARC
27349693 [7]	“Just-in-time” personal preparedness: downloads and usage patterns of the American Red Cross Hurricane application during Hurricane Sandy [7]	Observational	Hurricane Sandy	App users	Disaster response app	The number of downloads of the ARC hurricane app to access real-time information to assist with disaster preparation and recovery during Hurricane Sandy
27149308 [8]	Assessing American Red Cross First Aid mobile app user trends: Implications for resilience [8]	Descriptive study (secondary data analysis)	N/A	N/A	Disaster response app	ARC mobile app usage trends during a disaster
21466029 [9]	Emergent use of social media: a new age of opportunity for disaster resilience [9]	Systematic review	N/A	N/A	Social media	Survivor resilience
23202674 [10]	Assessing disaster preparedness among Latino migrant and seasonal farmworkers in eastern North Carolina [10]	Mixed methods	North Carolina	Latino migrant and seasonal farmworkers	ARC disaster preparedness	Civilian disaster preparedness
27170545 [11]	Mapping fires and American Red Cross aid using demographic indicators of vulnerability [11]	Cohort	N/A	Los Angeles County	ARC fire department	Demographic indicator of vulnerability, utilizing trends in ARC aid
18702270 [12]	Trial of a survey instrument to establish the hurricane preparedness of and medical impact on a vulnerable, older population [12]	Cross-sectional	FL Geriatrics Clinic	Elderly population	ARC disaster prep guidelines	Efficacy of guidelines
18217923 [13]	Enhancing community-based disaster preparedness with information technology [13]	pilot study of CBDP database	N/A	N/A	CBDP database	Efficacy of CBDP database
27863579 [14]	Evolution of a nursing model for identifying client needs in a disaster shelter: a case study with the American Red Cross [14]	Literature review	N/A	N/A	Screening in shelter	Efficacy of screening in the shelter
20301066 [15]	Public health preparedness of post-Katrina and Rita shelter health staff [15]	Cross-sectional	Hurricane Katrina, Hurricane Rita	Responders	ARC shelter health staff	Staff's public health awareness
19557953 [16]	Mississippi's infectious disease hotline: a surveillance and education model for future disasters [16]	Surveillance	Aftermath of Hurricane Katrina	N/A	Infectious disease surveillance system “hotline”	Number of patients in shelters who were diagnosed and were able to access more comprehensive healthcare; presence of significant casualties within shelters (death, significant infectious disease outbreaks)
24368053 [17]	Snapshot from Superstorm Sandy: American Red Cross mental health risk surveillance in lower New York State [17]	Descriptive study (cross-sectional)	Superstorm Sandy	Superstorm Sandy Survivors	Disaster operation sites (PsySTART)	Potential to address the mental health needs of survivors
23077267 [18]	The effectiveness of psychological first aid as a disaster intervention tool: research analysis of peer-reviewed literature from 1990-2010 [18]	Literature review	N/A	N/A	ARC psychological first aid	Effectiveness of psychological first aid
17295855 [19]	Vulnerable populations in an American Red Cross shelter after Hurricane Katrina [19]	Anecdote (mental health nurse)	Hurricane Katrina	N/A	ARC shelter resources	Vulnerable populations are at greater risk of harm
17583378 [20]	Burden of disease and health status among Hurricane Katrina-displaced persons in shelters: a population-based cluster sample [20]	Survey	Hurricane Katrina	Hurricane Katrina survivors in shelters	ARC shelter	Public health variables that make populations vulnerable
33332071 [21]	Pilot study on the experiences of hurricane shelter evacuees [21]	Survey (cross-sectional)	Post-Hurricane Florence	Hurricane Florence survivors in shelters	ARC shelter	Necessity of shelter post-Hurricane Florence (length of stay since the evacuation, number of shelters visited by the same party)
31221233 [22]	Disaster-related shelter surveillance during the Hurricane Harvey response – Texas 2017 [22]	Cohort	Hurricane Harvey	Hurricane Harvey survivors in shelters	Shelter	Demographic of shelter users during a disaster
11232378 [23]	Victim identification and family support in mass casualties: the Massachusetts model [23]	Pilot	Massachusetts	Responders, victims	Victim identification and family support	Efficacy of intervention
16712952 [24]	Mental health service use among American Red Cross disaster workers responding to the September 11, 2001 U.S. terrorist attacks [24]	Survey	N/A	Responders	Mental health services for ARC responders	Implied mental well-being of responders (use of mental health services)
17113159 [25]	Psychological distress among American Red Cross disaster workers responding to the terrorist attacks of September 11, 2001 [25]	Survey	N/A	Responders	General disaster response	Psychological distress of responders
15912717 [26]	Associations between alcohol use and PTSD symptoms among American Red Cross disaster relief workers responding to the 9/11/2001 attacks [26]	Survey	N/A	Responders	General disaster response	ARC responders’ well-being

Data from Table 1 has also been reorganized to view the accepted paper's characteristics based on different factors, such as study type, disaster (location), population studied, and interventions/outcomes studied, as shown in Table 2.

**Table 2:** Categorizing accepted citations based on the final paper characteristics

	Study type	Survey, pilot study, cohort study, systematic review, descriptive, literature review, anecdote, observational, mixed methods, cross-sectional, surveillance
	Disaster (location)	Hurricane Katrina, Hurricane Harvey, Hurricane Florence, Hurricane Sandy, Florida Geriatrics Clinic, Massachusetts, North Carolina, other locations
	Population studied	Evacuees, app users, Latino migrant/seasonal farmworkers, nursing programs, disaster responders, elderly population, other populations
Intervention studied	Disaster preparedness	Arc apps, social media, disaster prep guidelines, preparedness education (nursing)
	On-site disaster intervention	Shelter public health, triage systems, shelter resources, victim identification/family support
	Disaster recovery	General disaster response, mental health services for ARC responders
Outcome studied	Disaster preparedness	Accessibility of information, survivor disaster preparedness/resilience, preparedness education
	On-site disaster intervention	Necessity of shelter, vulnerable populations, efficacy of triage systems and other interventions, public health awareness
	Disaster recovery	Well-being of disaster responders

Results

Disaster Preparedness

Although ARC is known for its efforts in the aftermath of a harmful disaster, the organization also aims to train communities nationwide to become better prepared against future emergencies.

Professional collaborations: In addition to training its volunteers, ARC collaborates with other organizations, such as nursing programs, to ensure a well-rounded team of responders and to enhance disaster preparedness education. A 2017 study assessed the impact of this collaboration on pre-licensure BSN programs [5]. The partnership led to curriculum changes, including a "Pillowcase Project," where nursing students trained for six hours and then educated local children on disaster preparedness. This altered the nursing program's approach to Quality and Safety Education for Nurses domains like "patient-centered care" and "teamwork and collaboration" [5]. By expanding the number of healthcare workers who are prepared to assist ARC in its disaster efforts, the organization can provide a more holistic and stronger disaster response that prevents more survivors from being further harmed.

ARC apps and social media: Despite their extensive and educational in-person services, ARC has been making strides toward making information more accessible digitally. ARC offers free apps in English and Spanish, providing real-time disaster alerts, notifications, and information [6].

A study analyzed ARC’s hurricane-focused app usage during Hurricane Sandy in 2016 [7], showing a significant upsurge from 443 to 152,258 downloads during the crisis [6]. The study refers to this pattern specifically as “just-in-time” preparedness, or when individuals (or family units) begin to act when the “immediate threat risk is rapidly climbing” [7]. While the efficacy of just-in-time preparedness that ARC apps encourage cannot be ethically measured, it’s deemed that thousands were able to access vital information that reduced the stressors and suffering of navigating a natural disaster [7]. A similar trend occurred when another study analyzed ARC app user trends during tornadoes between 2013 and 2014 [8].

ARC also leverages social media for disaster preparedness, posting warnings and infographics on platforms like Instagram and X (formerly known as Twitter). Social media, when used to aid disaster preparedness, enables peer-to-peer interaction, contrasting with rigid centralized systems like television warnings [9]. Constant and consistent exchange of information enables users to obtain necessary information promptly without relying on a central, overwhelmed authority [9].

It is important to note that the ease of receiving and providing information can also lead to misinformation and might alter how effectively a community prepares for disaster. ARC may not be the only source broadcasting critical information about the disaster, which leaves room for more misinformation and poor disaster preparedness. Thus, apps like the one that ARC dedicated to disasters can still be beneficial to users, even if its algorithm does not allow for much public participation in contributing to disaster-related information. ARC employs strategies to prepare civilians, recognizing their benefits and shortcomings.

ARC and underprepared communities: Despite diverse types of responders recruited by ARC collaborations and ARC’s highly accessible information through digital resources, several populations continue to be underprepared for natural disasters [10-12].

In a study assessing disaster preparedness among Latino migrant and seasonal farmworkers (MSFW) in eastern Northern Carolina, MSFWs were both surveyed and in focus groups [10]. The findings indicated that while most farmworkers recognized the significance of natural disasters, particularly hurricanes, and expressed motivation to prepare, practical barriers hindered their efforts. Despite noting ARC as a trusted source of disaster-related information, many faced challenges such as limited resources and knowledge, with language, notably English proficiency, being a significant obstacle. Spanish-speaking MSFWs relied heavily on their bilingual community members to translate disaster warnings and alerts broadcast primarily in English [10].

Understanding which groups receive the most ARC aid during disasters is also essential to pinpoint areas lacking preparedness. This retrospective approach was conducted in a cohort study, where fires were mapped in Los Angeles County using “demographic indicators of vulnerability,” which were separated into age, income, and minority population [11]. Although demographic characteristics do not correlate to vulnerability in a disaster (for example, a wealthy person may not know how to evacuate a disaster despite their income), they are “strongly related to populations that will receive aid from ARC following fire” emergencies [11].

Even in disaster-experienced communities, some populations remain unprepared. A 2008 survey focusing on elderly patients found that many lacked essential elements of hurricane preparation, such as evacuation plans and emergency supplies [12].

Immediate Disaster Response

When disaster strikes, ARC promptly establishes temporary shelters for displaced survivors, playing a vital role in providing essential support and necessities through dedicated volunteers and staff who work tirelessly in the immediate aftermath.

Understanding the needs of evacuees and public health triages: ARC strives to address the diverse needs of individuals who seek assistance, especially during disasters when resources and responders may be limited.

A pilot study examined the effectiveness of a Community Disaster Information System (CDIS) implemented in conjunction with local ARC chapters to automate the coordination of disaster-related needs, including health services [13]. This system categorized services into ten areas, such as basic needs and health care, providing real-time updates via the Internet and offline access for use during connectivity disruptions. After two years, CDIS proved to be beneficial to organizations like ARC because the service allowed service providers to respond more efficiently to human needs [13].

Responders must also consider the overall community's health when mitigating disaster-related harm. A case study highlighted the evolution of ARC shelter triage, emphasizing public health nursing practices involving intake, cot-to-cot, and communications, maintaining health, independence, support, safety, self-determination, and transportation (CMIST) [14]. In registration intake, responders are guided to engage with distressed clients and allow clients themselves to share their immediate needs while in the shelter. Following intake, nurses undergo cot-to-cot procedures, where they physically check in with individuals and families, crucial for population surveillance to prevent disease or injury risks. CMIST considerations during cot-to-cot further aid in understanding evacuees' needs [14].

However, a study assessing public health preparedness among post-Katrina and Rita shelter health staff revealed a lack of preparedness in recognizing epidemiological patterns and conducting outbreak surveillance [15]. ARC was found to lack courses on public health preparedness, and only a minority of shelters had screening methods for evacuees upon arrival [15].

While the utilization of intake, cot-to-cot, and CMIST [14] may appear contradictory to the study revealing limited public health preparedness among responders [15], both studies hold some validity. Ultimately, the screening process mentioned lacked any criteria that suggested that a responder should conduct disease surveillance before an outbreak occurred, indicating a gap in ARC’s approach to public health.

To address this issue, Mississippi shelters during Hurricane Katrina implemented an "infectious disease hotline" as a surveillance system for health threats via phone calls [16]. This hotline connected responders and evacuees to public health staff, providing rapid feedback, guidance to shelter staff, and education on disaster-related infection outbreaks. This simple and specific surveillance proved essential for identifying potential outbreaks and caring for ill evacuees without medical professionals present [16].

ARC disaster response (mental health services): To enhance their disaster mental health response, ARC employs the Psychological Simple Triage and Rapid Treatment (PsySTART) [17]. This method involves communicating with survivors and recording their disaster exposure experiences, enabling shelters to efficiently provide targeted mental health support based on specific risk factors faced by evacuees. PsySTART offers a general understanding of ongoing mental health risks resulting from disasters, as demonstrated in a cross-sectional study by Schreiber et al. [17]. In the New York City region during Superstorm Sandy, PsySTART data revealed 18,823 disaster mental health contacts, identifying 17,979 reported risk factors, including issues like unlivable homes and mental health history [17]. Despite the need for a digital interface to improve PsySTART, the study highlighted its significance in accurately allocating resources and prioritizing the most vulnerable populations during the disaster response [17].

PsySTART enables responders to address mental health needs through psychological first aid (PFA) [18]. While not a substitute for professional therapy, PFA offers immediate care, comfort, and support to evacuees. A systematic review emphasized its role as an accessible intervention option, particularly in resource-limited and high-pressure environments where professional help is scarce [18].

Vulnerable populations of ARC shelters: The establishment of triage systems and mental health services in shelters offers relief to most evacuees, aiming to ensure that each individual's needs are met, even in chaotic situations. However, their effectiveness can vary depending on the scale of the disaster.

Hurricane Katrina displaced millions of households, overwhelming available resources and potentially leading to public health challenges [19]. Judith Saunders, an ARC psychiatric nurse volunteer, shared her experience in "Vulnerable Populations in an American Red Cross Shelter After Hurricane Katrina" [19]. The widespread damage caused resource shortages, including transportation, dietary options, and poor living conditions, exacerbating community health issues. Chronic diseases and stress among residents worsened due to the inherent uncertainty of the disaster [19]. Even efforts by the ARC to connect evacuees to recovery resources, such as housing, faced challenges due to long lines and limited capacities [19].

The destructive impact of Katrina also exposed health disparities within communities. A population-based cluster sample of Katrina evacuees revealed higher rates of health problems, including high blood pressure, compared to the national average [20]. This trend remains consistent with other medical problems reported, including high cholesterol and diabetes. Shelters are often considered a “last resort” when an individual has no alternative but to evacuate the crisis because they can only provide baseline aid and resources, implying discriminating accessibility to effective healthcare [20].

This pattern of disasters disproportionately affecting vulnerable populations extends beyond major events like Katrina. A study of a shelter during Hurricane Florence found that it primarily aided marginalized coastline residents, including the unemployed, those with no alternative housing options, and individuals needing medical care [21]. Shelter surveillance during Hurricane Harvey also revealed a significant number of client visits for treatable acute illnesses, emphasizing the need for more comprehensive health systems [22].

ARC family support services: In the face of emergencies, some individuals find themselves without shelter and support. During disasters with mass casualties, the ARC primarily focuses on providing family support services [23]. These services encompass contracting and consulting families, arranging for the return of personal belongings of the victims, and assisting in memorializing them. However, ARC does not conduct antemortem interviews, which are crucial for obtaining essential information about missing loved ones to aid in their identification. This responsibility falls under the purview of the chief medical officer. Instead, ARC assigns crisis counselors to families, providing emotional support both before and after the interviews. Unlike their authoritative and medical roles in shelters, ARC offers psychological support for the families involved in the identification process [23].

Long-Term Disaster Recovery

Well-being of ARC disaster responders: After being shut down, what remains of shelters are the staff and volunteers who dedicatedly ran and managed them. To comprehend the social impact of disasters on responders, the University of South Dakota conducted studies focused on analyzing the relationship between the 9/11 ARC disaster workers and certain mental health aspects [24-26].

In the first study, ARC disaster workers decreased their use of mental health services after 9/11. Fourteen percent of respondents used these services before 9/11, compared to 11% after 9/11. Researchers speculate that this trend is due to the experience gained from using mental health services before the event, which decreased the need to “seek treatment in response to the attacks.” Furthermore, researchers hint at potential “unique barriers” to mental health treatment during and after 9/11. The “unique barriers” were never identified nor elaborated on by the authors, but this could be a potential route of inquiry in the future [24].

In the second study, researchers determined that there was no meaningful relationship between the stressors ARC responders face and the amount of distress they are in. Although most disaster workers experienced some sort of distressing stimulus (including bomb threats), very few ARC workers reported post-traumatic stress disorder (PTSD) symptoms. The study attributes this finding to the sample, which contains mostly respondents who are older, well-educated, and married. All of these characteristics increase the surveyed individuals' access to mental health services and other support systems that decrease their post-traumatic stress symptoms [25].

In a related study, it was found that in addition to PTSD symptoms being low, ARC workers did not use alcohol, a substance often associated with PTSD, to cope with their harsh environment during 9/11 [26].

Limitations

The exclusion of gray literature, notably ARC's publications, in this review was a deliberate choice, driven by concerns over potential interpretation bias in their reports. As a result, the scoping review lacks insights into the social impact of ARC's interventions on disaster interventions. especially in recovery efforts. Additionally, it is essential to acknowledge the modest number of citations identified in this review, which stands in contrast to the citation count typically associated with a standard scoping review. Understanding the social impacts of ARC disaster interventions represents a relatively underexplored area within the academic discourse, marked by a scarcity of formally evaluated research papers. These limitations demonstrate the urgency for further research on this topic.

Another limitation of this review is that it was conducted without a registered review protocol, which may have influenced the selection and synthesis of studies. However, the author took steps to mitigate bias by adhering to a systematic and transparent methodology throughout the review process.

There is also a potential conflict of interest. Despite the author’s four-year volunteering period with the ARC, this scoping review maintains objectivity and adheres to established guidelines, relying solely on the analysis of available literature for its findings and interpretations. There was no financial support for this review.

Discussions

Increasing National Resilience Against Disasters

ARC appears to be moving in the correct direction regarding improving communities’ overall disaster preparedness when it comes to collaborating with organizations, especially those in the medical field, and establishing a digital presence. By diversifying emergency workers, ARC can ensure that evacuees and survivors can receive holistic and complete care during the ongoing crisis. Similarly, when ARC utilizes its digital apps and social media accounts, the organization diversifies the ways communities can receive critical information promptly. However, several reports have shown that some specific marginalized communities are still largely underprepared; factors such as language and age become obstacles when understanding what to do in a disaster. One interesting finding is that income and race do not correlate to an individual’s preparedness. Instead, the more aid the community receives from ARC after a specific disaster, the worse the community is at disaster preparedness.

We should clarify that we are not ignoring the fact that other disaster-oriented organizations are also at fault for not making disaster preparedness more accessible to underprepared communities. Instead, we are highlighting areas in which ARC can improve its disaster preparedness interventions.

In the future, ARC should strive to expand its collaborative network and tailor its support strategies to cater specifically to the needs of vulnerable communities. Additionally, leveraging data-driven insights can enhance resource allocation, with a particular focus on regions that have historically required substantial post-disaster assistance.

This paper acknowledges that ARC's initiatives extend beyond the discussed scope, including their educational programs and training in first aid and cardiopulmonary resuscitation CPR for volunteers. Further comprehensive research is needed to assess their social impact. Moreover, it's important to acknowledge that some of the studies discussed herein are geographically localized, limiting the generalizability of their findings.

Improving Future ARC Shelters

ARC has made commendable efforts in providing services within shelters during disasters, but there is room for improvement. While various databases and triage systems help responders assess public health needs, the absence of an infectious disease surveillance system and a knowledge gap among shelter responders in conducting such surveillance are evident. During Hurricane Katrina, Mississippi shelters successfully implemented an "infectious disease" hotline to address this gap, ensuring the safety of evacuees. In addition, the Red Cross offers several mental health services for evacuees, including a specialized PsySTART triage and PFA that allow survivors to get assistance even in the absence of a certified therapist. Some needs remain unseen despite these systems being used. Additionally, the population of shelters highlights health disparities within society. Furthermore, ARC's role extends beyond immediate shelter support, as it also assists families who have lost members during the crisis.

It's worth noting that most studies in this section focus on Hurricane Katrina, an event that had a disproportionate impact compared to other disasters, which may not directly translate to other disaster scenarios. Therefore, further research should investigate shelters of varying scale and circumstances.

While issues such as limited resources are challenging to anticipate due to the unpredictability of disaster scale, ARC can enhance public health within shelters by adopting a similar "hotline" approach that proved successful in identifying infectious disease risks during Hurricane Katrina.

ARC Responders and Disaster Recovery

Red Cross responders appear to be mentally resilient. Despite participating in an event that troubled the entirety of the country, responders appear to need fewer interventions to provide stability after 9/11.

Ignoring the self-reported publications by ARC, there are no formal research papers analyzing the social impacts of ARC’s disaster recovery aid for survivors. Without a comprehensive analysis of this subsection, a suggestion on how ARC can improve its recovery methods is inappropriate.

Lastly, it should be clarified that this paper does not ignore that disaster-oriented organizations are also at fault for not making disaster interventions more accessible to underprepared communities. Nor does it aim to claim that ARC is not an organization to be trusted with disaster-related emergencies. To reiterate the objective of this paper, the authors aim to first identify the social impacts of ARC's disaster interventions and then pinpoint areas in which ARC can improve its efforts.

## Conclusions

The purpose of the study was to survey and investigate the social impacts of ARC’s disaster interventions. “Social impacts” in this review were measured by the accessibility of ARC disaster intervention resources to especially vulnerable resources. This investigation revealed that the ARC generally attempts to confront the health needs of communities that are aided by it before, during, or after the disaster has occurred. However, this review identifies several gaps in their disaster interventions, such as lacking language options for disaster preparedness information and inconsistent public health knowledge across ARC shelter staff. Taken together, these results suggest that ARC is moving in the correct direction in reducing the harm disasters cause to communities.
