# Correction: Social Impacts of the American Red Cross (ARC) Disaster Interventions: A Scoping Review

**DOI:** 10.7759/cureus.c345

**Published:** 2025-10-03

**Authors:** Mishayla A Harve, Dan Li

**Affiliations:** 1 Ivan Allen College of Liberal Arts, Georgia Institute of Technology, Atlanta, USA; 2 College of Medicine, Yale University, New Haven, USA

This article has been corrected to fix the following typos in the paragraph immediately preceding Figure 1: "**Sixty-one** papers passed the first stage of screening. Of the **61** papers, 22 passed the second stage of screening." corrected to "**Sixty** papers passed the first stage of screening. Of the **60** papers, 22 passed the second stage of screening."

